# Expression of AR-V7 (Androgen Receptor Variant 7) Protein in Granular Cytoplasmic Structures Is an Independent Prognostic Factor in Prostate Cancer Patients

**DOI:** 10.3390/cancers12092639

**Published:** 2020-09-16

**Authors:** Paul König, Markus Eckstein, Rudolf Jung, Amer Abdulrahman, Juan Guzman, Katrin Weigelt, Ginette Serrero, Jun Hayashi, Carol Geppert, Robert Stöhr, Arndt Hartmann, Bernd Wullich, Sven Wach, Helge Taubert, Verena Lieb

**Affiliations:** 1 Department of Urology and Pediatric Urology, Universitätsklinikum Erlangen, Friedrich-Alexander-Universität Erlangen-Nürnberg, 91054 Erlangen, Germany; paul.koenig@fau.de (P.K.); Amer.Abdulrahman@uk-erlangen.de (A.A.); Juan.Guzman@uk-erlangen.de (J.G.); Katrin.Weigelt@uk-erlangen.de (K.W.); Bernd.Wullich@uk-erlangen.de (B.W.); sven.wach@uk-erlangen.de (S.W.); Verena.Lieb@uk-erlangen.de (V.L.); 2 Department of Pathology, Universitätsklinikum Erlangen, Friedrich-Alexander-Universität Erlangen-Nürnberg, 91054 Erlangen, Germany; Markus.Eckstein@uk-erlangen.de (M.E.); Rudolf.Jung@uk-erlangen.de (R.J.); carol.geppert@uk-erlangen.de (C.G.); robert.stoehr@uk-erlangen.de (R.S.); arndt.hartmann@uk-erlangen.de (A.H.); 3 A&G Pharmaceutical Inc., Columbia, MD 21045, USA; gserrero@agpharma.com; 4 Program in Oncology, University of Maryland Greenebaum Comprehensive Cancer Center, Baltimore, MD 21201, USA; jhayashi@precisionantibody.com; 5 Precision Antibody, Columbia, MD 21045, USA

**Keywords:** AR, AR-V7, prostate cancer, Gleason score, perineural invasion, relapse-free survival, prognosis, RFS

## Abstract

**Simple Summary:**

The expression of the androgen receptor (AR) and its splice variant AR-V7 is crucial for prostate cancer (PCa) biology. An immunohistochemical staining was performed on a tissue microarray with specimens from 410 PCa patients. AR staining, neither in the nucleus nor in the cytoplasm was associated with prognosis. AR-V7 staining of the general cytoplasm was associated with a shorter relapse free survival (RFS), whereas AR-V7 staining of cytoplasmic granules was associated with a longer RFS. Further subgroup stratification for AR-V7 granular staining revealed it as an independent prognostic factor in younger patients (age ≤ 65), patients with negative CK20 staining and patients with perineural invasion. Altogether, AR-V7 protein detected in granular cytoplasmic structures is an independent prognostic factor for RFS in PCa patients.

**Abstract:**

Prostate cancer (PCa) is the second most common cancer, causing morbidity and mortality among men world-wide. The expression of the androgen receptor (AR) and its splice variants is a crucial factor of prostate cancer biology that has not been comprehensively studied in PCa tumors. The aim of this study was to characterize the protein expression of the AR and its splice variant, AR-V7, and their subcellular distributions in PCa by immunohistochemistry and to correlate the results to the clinicopathological data and prognosis. Immunohistochemical staining for AR and AR-V7 was performed on a tissue microarray (TMA) with specimens from 410 PCa patients using an immunoreactive score (IRS) or only the percentage of AR-V7 staining in cytoplasmic granules. Nuclear or cytoplasmic AR staining was not associated with prognosis. AR-V7 staining was only occasionally observed in the nucleus. However, AR-V7 staining in the cytoplasm or in cytoplasmic granules was associated with relapse-free survival (RFS). AR-V7 staining of the cytoplasm was associated with a shorter RFS, whereas AR-V7 staining of cytoplasmic granules was associated with a longer RFS. In a multivariate Cox’s regression analysis, only negative (<5%) AR-V7 staining of cytoplasmic granules remained an independent prognostic factor for RFS (HR = 5.3; *p* = 0.006). In a further subgroup analysis by multivariate Cox’s regression analysis, AR-V7 was an independent prognostic factor in the following groups: age ≤ 65 (HR = 9.7; *p* = 0.029), negative CK20 staining (HR = 7.0; *p* = 0.008), and positive perineural invasion (HR = 3.7; *p* = 0.034). Altogether, AR-V7 protein in granular cytoplasmic structures is an independent prognostic factor for RFS in PCa patients.

## 1. Introduction

Prostate cancer (PCa) represents the second most common cancer and the fifth most common cause of cancer-associated death in men globally [1]. PCa is a group of histologically and molecularly heterogeneous diseases with variable clinical courses. In its early, localized stages, PCa is usually treated with curative intent by local therapy, such as radical prostatectomy, external beam radiation therapy, or brachytherapy. Remarkably, PCa is strictly dependent on androgen receptor (AR) signaling in all stages of disease, except for neuroendocrine differentiation. Therefore, androgen deprivation therapies (ADT) aimed at disrupting AR signaling by surgical or chemical castration, combined in selected cases with antiandrogens, are the standard care for locally advanced or metastatic disease. However, although the disease may be controlled for several years, the benefit of ADT is only temporary. A progression of PCa towards castration-resistant PCa (CRPC) or its metastatic form (mCRPC) is a major cause of morbidity and mortality. In addition to the PCa classification by the World Health Organization (WHO) [2], molecular classification of different cancers, including PCa, at the RNA level revealed a luminal-like and basal-like transcript pattern. This classification is associated with prognosis and response to androgen deprivation therapy in PCa [3,4]. Here, we applied cytokeratin 20 (CK20) as a proxy marker for a luminal-like transcript pattern. However, such a classification based on protein expression does not yet exist for PCa. There are several biomarker assays on the market [5,6,7,8], but they are not yet widely used in urologic practice.

Based on the dependency of PCa on AR signaling, most therapeutic approaches affect the androgen receptor itself, androgen biosynthesis and/or the interaction of the AR with androgens. As a consequence, the tumor develops therapeutic resistance to AR-targeted therapies due to AR mutations, AR amplification or AR splice variants [9,10,11]. Among the more than 30 AR splice variants [12], AR-V7 appears to be the most clinically relevant variant [11]. Its protein expression in tumor tissue or circulating tumor cells (CTCs) has been associated with shorter overall survival (OS), disease-specific survival (DSS) and/or recurrence-free survival (RFS) [13,14,15]. However, AR-V7 protein detection is mostly focused on nuclear staining, while cytoplasmic staining patterns have not been further evaluated. There is only one article that described an association of cytoplasmic AR-V7 staining with shorter RFS [16].

The aim of the present study was to examine AR and AR-V7 protein expression and their subcellular distributions in PCa and to correlate the findings with clinicopathological and prognostic data.

## 2. Results

### 2.1. AR and AR-V7 Expression and Correlation with Clinicopathological Parameters and the Expression of Selected Proteins

**Figure 1:**
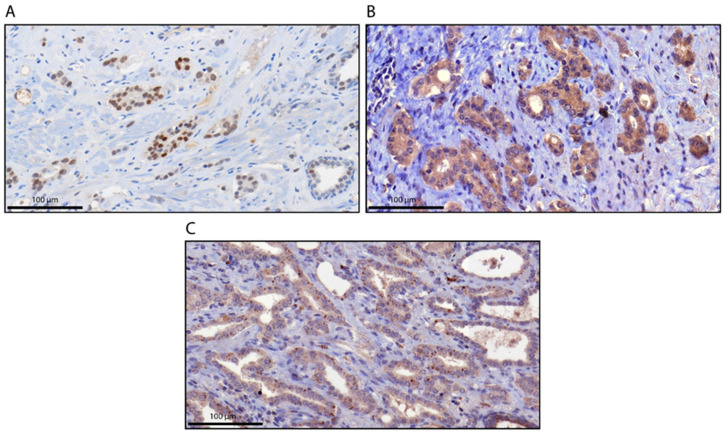
Immunohistochemical staining for androgen receptor (AR) and AR-V7. (**A**) AR staining in the nucleus (immunoreactive score (IRS) = 9; 70% strong); (**B**) AR-V7 staining in the cytoplasm (IRS = 12; >95% strong). (**C**) AR-V7 staining in cytoplasmic granules (>5%). The scale bar represents 100 µm.

A TMA containing prostate tumor sections from a cohort of 410 PCa patients was evaluated for AR and AR-V7 protein expression by immunohistochemistry (IHC) using an AR-V7 monoclonal antibody as described in the methods section. AR staining was detected in the nucleus and cytoplasm, whereas AR-V7 expression was observed in the cytoplasm (cytoplasmic) or in distinct granular structures in the cytoplasm (granular), but only occasionally in the nucleus, by IHC. Figure 1 shows representative photomicrographs of AR- and AR-V7-stained specimens. Expression was assessed with an IRS for AR in the cytoplasm/nucleus and for AR-V7 in the cytoplasm as described in the Methods section. In addition, the percentage of AR-V7 stained granules was determined. The threshold for positivity was set at an IRS of 2 for cytoplasmic/nuclear AR and cytoplasmic AR-V7, with negative groups (IRS ≤ 2) and positive groups (IRS > 2), and the threshold was set at 5% for granular AR-V7 staining (AR-7 < 5% vs. AR-V7 ≥ 5%).

The staining distributions for AR and AR-V7 were as follows: For AR cytoplasmic staining, 282 cases (68.8%) were negative, and 127 cases (31.0%) were positive. For AR nuclear staining, 96 cases (23.4%) were negative, and 314 cases (76.6%) were positive. For AR-V7 cytoplasmic staining, 59 cases (14.4%) were negative, and 347 cases (84.6%) were positive. For AR-V7 granular staining, 261 cases (63.7%) were negative, and 144 cases (35.1%) were positive (Table 1; Table S1). However, AR-V7 nuclear staining occurred only in 25 cases (6.2%).

AR cytoplasmic staining was directly (positively) correlated with perineural invasion (Pn) (r_s_ = 0.142; *p* = 0.005), prostatectomy Gleason sum (GS) (r_s_ = 0.146; *p* = 0.004), relapse occurrence (RFS) (r_s_ = 0.122; *p* = 0.014), pathological tumor stage (pT) (r_s_ = 0.160; *p* = 0.001), CK20 staining (r_s_ = 0.154; *p* = 0.002), AR nuclear staining (r_s_ = 0.652; *p* < 0.001), and AR-V7 cytoplasmic staining (r_s_ = 0.482; *p* < 0.001), but it was not inversely or negatively correlated with any clinicopathological or molecular factor (Table S2).

AR nuclear staining was positively correlated with Pn (r_s_ = 0.112; *p* = 0.028), pT (r_s_ = 0.102; *p* = 0.038), CK20 staining (r_s_ = 0.222; *p* < 0.001), AR cytoplasmic staining (r_s_ = 0.652; *p* < 0.001), and AR-V7 cytoplasmic staining (r_s_ = 0.401; *p* < 0.001). However, it was negatively correlated with AR-V7 granular staining (r_s_ = -0.109; *p* = 0.028; Table S2).

AR-V7 cytoplasmic staining was positively correlated with Pn (r_s_ = 0.271; *p* < 0.001), prostatectomy GS (r_s_ = 0.167; *p* = 0.001), pT (r_s_ = 0.152; *p* = 0.002), CK20 staining (r_s_ = 0.116; *p* = 0.019), AR nuclear staining (r_s_ = 0.401; *p* < 0.001), and AR cytoplasmic staining (r_s_ = 0.482; *p* < 0.001). It was negatively correlated with AR-V7 granular staining (r_s_ = −0.173; *p* < 0.001; Table S2).

AR-V7 granular staining showed no positive correlation with any clinicopathological or molecular factor, but it was negatively correlated with Pn (r_s_ = −0.187; *p* < 0.001), prostatectomy GS (r_s_ = −0.147; *p* = 0.004), RFS (r_s_ = −0.204; *p* < 0.001), pT (r_s_ = −0.169; *p* = 0.001), metastasis occurrence (r_s_ = −0.173; *p* < 0.001), AR nuclear staining (r_s_ = −0.109; *p* = 0.028), and AR-V7 cytoplasmic staining (r_s_ = −0.173; *p* < 0.001; Table S2).

### 2.2. Specificity of AR-V7 Staining

**Figure 2:**
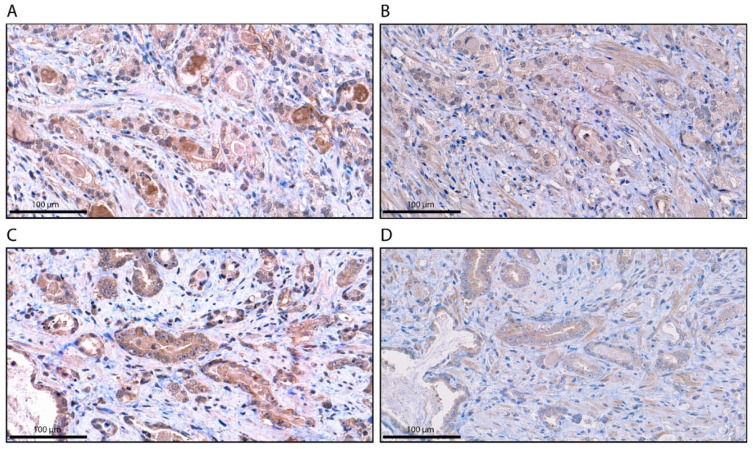
Immunohistochemical staining for AR-V7 without and with blocking peptide (BP). (**A**) AR-V7 staining in the cytoplasm without the BP; (**B**) AR-V7 staining in the cytoplasm with the BP. (**C**) AR-V7 staining in cytoplasmic granules without the BP; (**D**) AR-V7 staining in cytoplasmic granules with the BP. The scale bar represents 100 µm.

To confirm the specificity of AR-V7 staining for the different localizations, a synthetic AR-V7 blocking peptide comprising the 9-C-terminal amino acids (CKHLKMTRP) encoded by the cryptic exon 3 (CE3) of AR-V7, that competes with the AR-V7 antibody was applied as previously described [17]. Application of the synthetic AR-V7 blocking peptide resulted in a loss of AR-V7 staining in the cytoplasm, nucleus and granular structures (Figure 2). Therefore, AR-V7 staining at all localizations was specific.

### 2.3. Association of AR and AR-V7 Protein Expression with Survival

The association of AR and AR-V7 staining in the 410 PCa tumor samples with patient survival was examined by Kaplan-Meier analysis. AR staining in the nucleus or in the cytoplasm was not associated with OS, DSS, nor RFS. In addition, both AR-V7 staining in the cytoplasm and in the cytoplasmic granules were not associated with OS or DSS.

**Figure 3:**
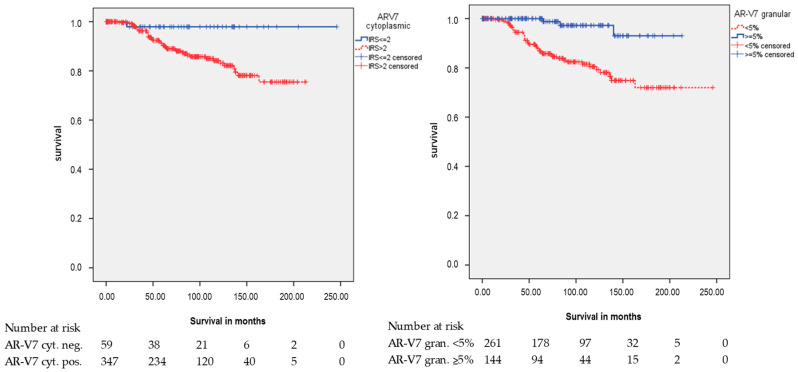
Kaplan-Meier analysis: Association of AR-V7 cytoplasmic staining and AR-V7 cytoplasmic granular staining with relapse-free survival (RFS). AR-V7 cytoplasmic and AR-V7 cytoplasmic granular protein expression was associated with RFS (*p* = 0.027 and *p* < 0.001; all log rank test).

However, both AR-V7 staining patterns were significantly associated with RFS but had opposite correlations (Figure 3). In Kaplan-Meier analysis, positive cytoplasmic AR-V7 staining was associated with a shorter RFS of 182.5 months, whereas patients with a negative staining had a mean RFS of 241.3 months (*p* = 0.027; Table 2). Univariate Cox’s regression analysis (Table S3) revealed that cytoplasmic AR-V7 positivity had a trend towards being associated with a 6.8-fold risk of relapse occurrence (*p* = 0.057).

Since AR cytoplasmic and AR-V7 cytoplasmic staining are related, we were interested if their combination could be associated with prognosis. Combination of both staining patterns resulting in four groups, showed no significant differences in OS (*p* = 0.263), DSS (*p* = 0.473), nor RFS (*p* = 0.164) (data not shown). However, when we considered only the best (both negative) and the worst prognosis groups (both positive), there was a significant difference in RFS (*p* = 0.025; log rank test, Figure S1) but again not in OS (*p* = 0.860) or DSS (*p* = 0.309). Univariate Cox’s regression analysis revealed that the combination of cytoplasmic AR and cytoplasmic AR-V7 positivity had the same trend as the single cytoplasmic AR-V7 positivity towards being associated with a 6.8-fold risk of relapse occurrence (*p* = 0.061; data not shown).

In Kaplan-Meier analysis, negative granular AR-V7 staining was associated with a shorter RFS (*p* < 0.001; Table 2). Univariate Cox’s regression analysis revealed that cytoplasmic granular AR-V7 negativity was associated with a 7.2-fold risk of relapse occurrence (*p* = 0.001; Table 3).

Before performing multivariate analysis, we tested the clinicopathological factors that were mainly correlated with AR-V7 staining in univariate Cox’s regression analysis for their association with RFS. Patient age and CK20 staining were not significantly associated with RFS. However, pT, prostatectomy GS and Pn were significantly associated with RFS (Table S3).

Multivariate Cox’s regression analysis, adjusted for pT, prostatectomy GS and Pn, revealed no association between cytoplasmic AR-V7 positivity and RFS (HR = 4.6; *p* = 0.133; data not shown).

Multivariate Cox’s regression analysis (adjusted for pT, prostatectomy GS and Pn) showed that negative granular AR-V7 staining was an independent prognostic factor for RFS (HR = 5.3; *p* = 0.006; Table 3).

Next, we performed a subgroup analysis for pT, prostatectomy GS, age at diagnosis, Pn status, and CK20 staining.

### 2.4. Subgroup Analysis for AR-V7 Cytoplasmic and AR-V7 Granular Staining

Subgroup analysis by Kaplan-Meier analysis and univariate/multivariate Cox’s regression analysis did not reveal a significant association between AR-V7 cytoplasmic staining and RFS in any subgroup (Table 2 and data not shown).

**Figure 4:**
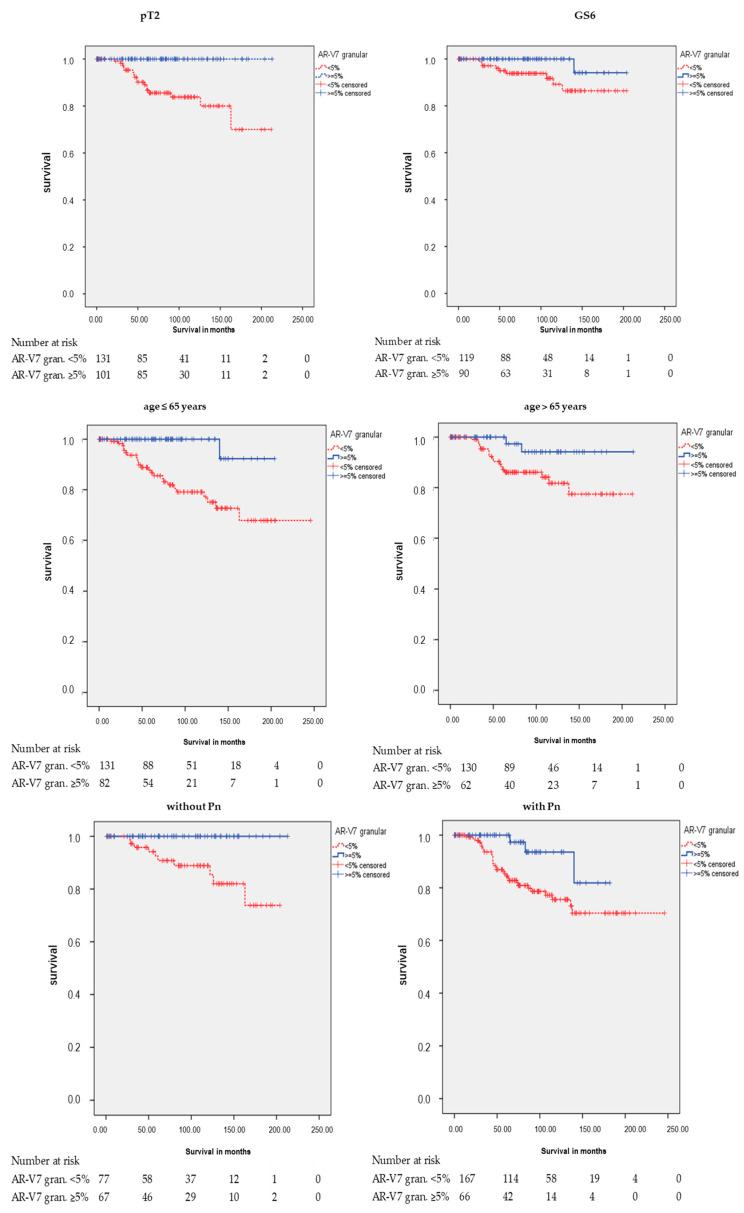

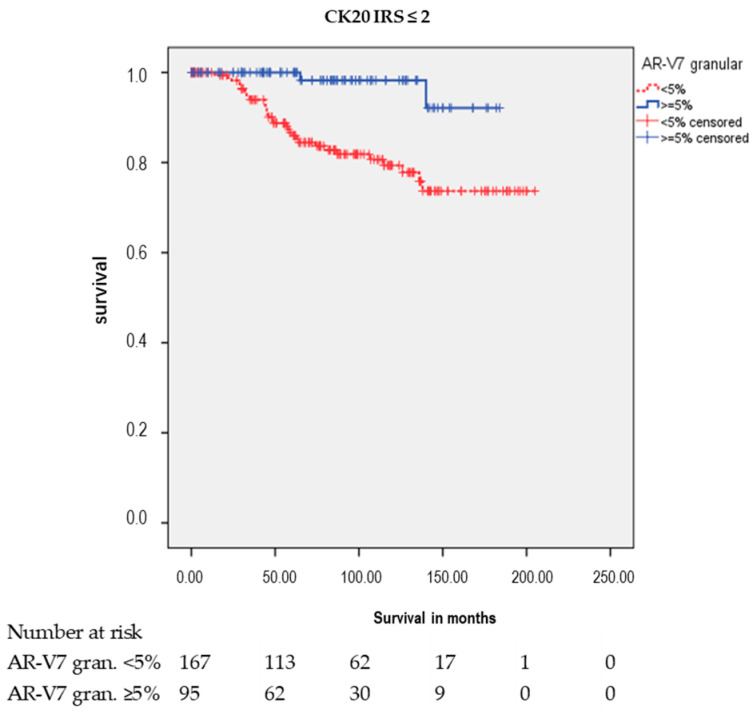
Kaplan-Meier analysis: Association of AR-V7 cytoplasmic granular staining with RFS in subgroups. Positive AR-V7 cytoplasmic granular staining (≥5%) was associated with longer RFS in the subgroups pathological tumor stage 2 (pT2: *p* < 0.001), Gleason score 6 (GS6: *p* = 0.040), age at diagnosis ≤ 65 years (*p* = 0.001), age at diagnosis > 65 years (*p* = 0.045), without Pn (*p* = 0.005), with Pn (*p* = 0.018), and negative CK20 staining (IRS ≤ 2: *p* = 0.001).

However, there were significant associations between AR-V7 granular staining and RFS in different subgroups (Table 2 and Table 3; Figure 4). Positive granular AR-V7 staining was associated with longer RFS in the subgroups pT2 (*p* < 0.001), GS 6 (*p* = 0.040), age at diagnosis ≤ 65 years (*p* = 0.001), age at diagnosis > 65 years (*p* = 0.045), without Pn (*p* = 0.005), with Pn (*p* = 0.018), and negative CK20 staining (IRS ≤ 2; *p* = 0.001) in the Kaplan-Meier analysis (Table 2). In the multivariate Cox’s regression analysis (adjusted for pT, prostatectomy GS, and Pn), AR-V7 granular staining remained an independent prognostic factor for longer RFS in the following groups: age ≤ 65 (HR = 9.7; *p* = 0.029), negative CK20 staining (HR = 7.0; *p* = 0.008), and in the group with Pn (HR = 3.7; *p* = 0.034; here, multivariate Cox’s regression analysis adjusted for pT and prostatectomy GS) (Table 3).

### 2.5. Characterization of the AR-V7 Positively Stained Granular Structures

**Figure 5:**
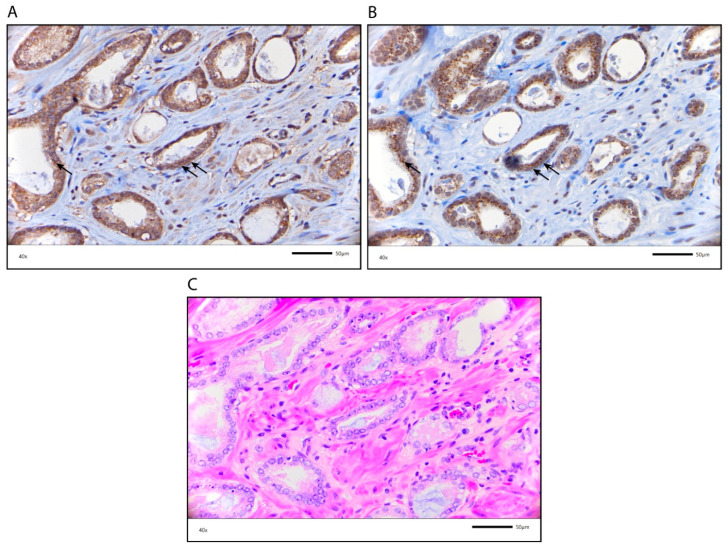
Similar localization of AR-V7 and giantin, a Golgi apparatus marker. (**A**) AR-V7 staining in cytoplasmic granules; (**B**) Giantin staining in the Golgi apparatus. Arrows point to similar localization of AR-V7 and giantin. (**C**) HE staining of the PCa specimen (corresponding to staining in (**A**,**B**)). The scale bar represents 50 µm.

Next, we examined which granular structures were stained for AR-V7. Since the structures resembled the Golgi apparatus, we stained the PCa TMA for GOLGB1 (also known as giantin, macrogolgin, and GCP372), a major protein of the Golgi apparatus. Interestingly, we observed similar granular structures stained by both AR-V7 and giantin (Figure 5). Although double staining for both proteins was not performed, their coinciding staining pattern suggests that AR-V7 is localized in the Golgi apparatus.

## 3. Discussion

The AR is the most important hormone receptor in PCa, and its splice variants, mainly AR-V7, can participate in endocrine resistance [12]. Here, the protein expression of AR and AR-V7 in tumors from 410 PCa patients was analyzed and correlated with clinicopathological parameters and survival data. AR staining was detected in the nucleus and in the cytoplasm, whereas AR-V7 staining was seen in the cytoplasm as general staining and/or localized in cytoplasmic granule-like structures. Nuclear staining for AR-V7 was only detected in a minority of cases (25/406 cases), and it did not show an association with OS, DSS, nor RFS. Most AR-V7 studies in PCa reported and characterized AR-V7 staining in the nucleus but did not describe AR-V7 staining in the cytoplasm. Remarkably, these studies detected more AR-V7 nuclear staining, but they analyzed PCa patients who had been treated with neoadjuvant hormones or who had highly aggressive PCa [11,13]. We studied primary PCa, and a small number of cases expressing nuclear AR-V7 can be expected. However, one study reported general cytoplasmic staining of AR-V7 and its relationship to prognosis, i.e., RFS [16]. Distinct cytoplasmic granular AR-V7 staining is described for the first time in this study.

By correlating AR cytoplasmic, AR nuclear, and AR-V7 cytoplasmic staining with clinicopathological data and with each other, we found a positive correlation with perineural invasion (Pn), pathological tumor stage (pT), and CK20 staining and between these three staining patterns (AR cytoplasmic, AR nuclear, and AR-V7 cytoplasmic). Additionally, AR cytoplasmic and AR-V7 cytoplasmic staining was negatively correlated with the Gleason sum (GS) at prostatectomy, whereas AR-V7 cytoplasmic staining was associated with relapse occurrence. AR-V7 granular staining showed the opposite result, i.e., a negative correlation with the clinicopathological parameters Pn, prostatectomy GS, relapse occurrence, pT, metastasis occurrence, and with AR nuclear and AR-V7 cytoplasmic staining.

In our survival analysis, AR staining in the nucleus or in the cytoplasm was not associated with OS, DSS, nor RFS. In addition, AR-V7 staining in both the cytoplasm and the cytoplasmic granules was not associated with OS or DSS. However, positive AR-V7 staining in the cytoplasm was a negative prognostic marker for RFS in univariate analysis, in agreement with the multivariate analysis result by Guo et al. [16]. Conversely, in our multivariate analysis, cytoplasmic AR-V7 staining did not appear to be an independent prognostic marker. This difference could be explained by the fact that, in addition to Gleason sum and pT, Guo et al. adjusted their analysis for preoperative PSA levels and surgical margins, while we adjusted for Pn, which is strongly associated with shorter RFS, as reported by Katz et al. [18] and this study.

Based on the assumption that only nuclear AR-V7 can affect gene transcription, several studies scored only nuclear AR-V7 expression and disregarded cytoplasmic staining in their experiments [13]. Increased AR-V7 nuclear staining was associated with poor prognosis [13,14,19]. However, Chen et al. recently showed that the association for RFS with poor prognosis was only for a selected high-risk PCa cohort but not for an unselected cohort [13]. Altogether, we suggest that, in addition to nuclear AR-V7 protein staining, cytoplasmic AR-V7 protein staining should be included in further analyses to characterize its prognostic ability for PCa patients.

We showed, for the first time, that AR-V7 granular staining (≥5%) is an independent prognostic biomarker for longer RFS in PCa patients. Next, we were interested in whether this prognostic effect could be further stratified into different clinicopathological subgroups. We found that AR-V7 granular staining (≥ 5%) was an independent prognostic marker for RFS in the subgroups age ≤ 65 years, patients with Pn and patients with negative CK20 staining (IRS ≤ 2) but was not in the groups age > 65 years and patients with positive CK20 staining (IRS > 2). Recently, we showed that another marker, i.e., progranulin (GP88) protein expression, was associated with a shorter RFS in younger PCa patients. Since there is an increase in early-onset PCa in Europe and America [20], the identification of prognostic markers in this age group of PCa patients is of special interest. However, GP88 expression and AR-V7 granular staining were not correlated with each other (data not shown). We found a correlation between AR cytoplasmic/AR nuclear staining, AR-V7 cytoplasmic staining and CK20 protein expression. Cancers, including PCa, expressing cytokeratin 20 RNA can display a better prognosis than cancers with low cytokeratin transcript levels that belong to the basal-like subtype [3]. However, at the protein level, cytokeratin 20 is not used routinely as a pathological marker for luminal-like subtypes in PCa. No difference in RFS between CK20-positive and CK20-negative PCa tumors was observed. Further studies are necessary to investigate the relationship between CK20 protein and AR/AR-V7 protein expression in PCa.

Next, we were interested in why AR-V7 granular staining could be associated with a longer RFS. AR-V7 participates in endocrine resistance [12]. Therefore, it was unexpected to see its association with a longer RFS. It is possible that AR-V7 is involved in a protein degradation process rather than having a direct positive effect on RFS. The granular staining resembles the granular distribution of Golgi proteins. One of the major Golgi proteins is GOLGB1/giantin [21,22], which also shows a granular staining pattern (https://www.proteinatlas.org/ENSG00000173230-GOLGB1/pathology). We think AR-V7 detected as granular staining means that AR-V7 is not active. We hypothesize that granular AR-V7 is involved in a protein degradation process in the Golgi apparatus. In this way, AR-V7 cannot function as transcription factor and this may slow down tumor growth and tumor development resulting in a longer RFS.

We showed in this study that the granular staining patterns of AR-V7 and giantin are very similar, suggesting that granular AR-V7 is located in the Golgi apparatus. However, what function could AR-V7 have in the Golgi apparatus? There are several mechanisms for cellular proteostasis, including protein-degradation mechanisms (reviewed in Reference [23]). Recently, in yeast, a new endosome and Golgi-associated degradation pathway (EGAD) was identified that functions mostly for the degradation of membrane proteins [24]. It is tempting to speculate that such a mechanism may also exist in humans and could play a role in general protein degradation. However, further studies are necessary to test this hypothesis.

## 4. Materials and Methods

### 4.1. Patients and Tumor Samples

The tissue microarrays (TMAs) contained consecutively collected, formalin-fixed and paraffin embedded tumor samples of 410 PCa patients. They were diagnosed in the Department of Pathology, University Hospital Erlangen between 1999 and 2010. The tumors were collected from radical prostatectomy specimens, and the follow-up time from date of diagnosis ranged from 0 to 246 months (median 95 months). For OS, the observation time ends with the death of the patient (any reason = event, i.e., either disease-specific or disease-unspecific death), all other patients that were alive at their last visit in the observation time are considered as alive (no event). For DSS, the observation ends either with the tumor-specific death (=event); any other reason for death or patients that are still alive in the follow up time are considered as alive (no event). For RFS, the observation time ends either with a recurrence (=event), all patients who did not develop a recurrence within the observation time are considered as recurrence-free (=no event). The tumor histology was reviewed by experienced uropathologists (AH and ME). All procedures were performed in accordance with the ethical standards established in the 1964 Declaration of Helsinki and its later amendments. All patients starting in 2008 provided written informed consent. For samples collected before 2008, the Ethics Committee in Erlangen waived the need for informed individual consent. The study was approved by the Ethics Committee of the University Hospital Erlangen (No. 3755). Since archival tissue was studied several years after resection, the analysis of AR and AR-V7 protein expression was retrospective without affecting clinical decisions. Clinicopathological data of the patients are shown in Table 1. The PSA level was determined prior to prostatectomy.

### 4.2. Immunohistochemistry

For analysis of AR and CK20 protein expression, IHC staining was performed on a fully automated Ventana Benchmark Ultra autostainer (Ventana, Tucson, AZ, USA). The following antibodies were applied: AR expression was analyzed with a monoclonal mouse AR antibody (Dako, Hamburg, Germany, Clone AR411, dilution 1:50) and CK20 expression was studied with a monoclonal mouse CK20 antibody (Dako, Clone Ks 20.8, dilution 1:50) by a routine staining procedure as previously described [25]. Briefly, sections were deparaffinized, and antigen retrieval was performed by heating the sections in a pH 8.4 Tris/borate/EDTA solution (Ventana). Endogenous peroxidase was blocked with 1% H_2_O_2_. Visualization of bound antibody was performed using the ultraVIEW TM DAB system (Ventana). All sections were counterstained with hematoxylin II/Mayer’s hematoxylin (Ventana).

For the AR-V7 protein expression study, a manual immunohistochemistry (IHC) protocol was applied as previously described [26]. A primary antibody against AR-V7 (monoclonal mouse anti-AR-V7 antibody, dilution 1:40; Cat. No. AG10008; Precision Antibody, Columbia, MD, USA) was applied for 30 min. The stained specimens were viewed at an objective magnification of 100× and 200×.

The expression of AR (nucleus/cytoplasm), AR-V7 (cytoplasm) and CK20 (cytoplasm) was detected by assessing the percentage of stained tumor cells and the staining intensity semiquantitatively. The percentage of positive cells was scored as follows: 1, 1–9% positive cells; 2, 10–50%; 3, 51–80%; and 4, >80% positive cells. Staining intensity was scored as 0, negative; 1, weak; 2, moderate; and 3, strong. The immunoreactive score (IRS) was calculated as the product of staining percentage and staining intensity, resulting in an immunoreactive score (IRS) from 0 to 12 [27]. Negative control slides without the addition of primary antibody were included for each staining experiment. For the IHC analysis, the patients were grouped by IRS ≤ 2 and IRS > 2 for AR, AR-V7, and CK20 staining. AR-V7 in the granular structures was reported in percentage per tumor cells. We observed AR-V7 in granular structures only when it was also detected in the cytoplasm. Slides were scanned with a P250 slide scanner (3DHistech, Budapest, Hungary) and analyzed using CaseViewer2.0 (3DHistech).

### 4.3. Statistical Analyses

The correlations between the IHC scores and clinicopathological data were calculated using Spearman’s bivariate correlation or the chi^2^-test. The associations of AR and AR-V7 expression with overall survival (OS), disease-specific survival (DSS), and relapse-free survival (RFS) were determined by univariate (Kaplan–Meier analysis and Cox’s regression hazard models) and multivariate (Cox’s regression hazard models, adjusted for pT, prostatectomy GS and Pn) analyses. A *p*-value < 0.05 was considered statistically significant. The statistical analyses were performed with the SPSS 21.0 software package (SPSS Inc., Chicago, IL, USA).

## 5. Conclusions

AR-V7 staining in the cytoplasm or in cytoplasmic granules was associated with relapse-free survival (RFS) in the Kaplan-Meier analysis. However, AR-V7 cytoplasmic positive staining was associated with a shorter RFS, and AR-V7 granular staining was associated with a longer RFS. In a multivariate Cox’s regression analysis, negative (< 5%) AR-V7 granular staining remained an independent prognostic factor for RFS. Further subgroup analysis revealed that AR-V7 granular staining was an independent prognostic factor in the following groups: younger patients (age ≤ 65), patients with negative CK20 staining and patients with perineural invasion. Altogether, AR-V7 protein in granular cytoplasmic structures appears to be an independent prognostic factor for RFS in PCa patients.

## Figures and Tables

**Table 1:** Clinicopathological and immunohistochemical data for the prostate cancer (PCa) patients.

All PCa Patients	N (%)
	410
Age, median in years (range) (IQR)	65 (45−83) (61−69)
Pathological tumor stage (pT)	
pT2	236 (57.6)
pT3	144 (35.1)
pT4	30 (7.3)
Gleason score (GS) at prostatectomy	
GS 6	213 (52.0)
GS 7a	78 (19.0)
GS 7b	30 (7.3)
GS 8	27 (6.6)
GS 9−10	34 (8.3)
GS unknown	28 (6.8)
PSA (ng/mL) at prostatectomy (median) (IQR)	(3.86) (0.99−7.59)
< 4 ng/mL	174 (42.4)
≥ 4 ng/mL	171 (41.7)
Unknown	65 (15.9)
Perineural invasion	
No	147 (35.8)
Yes	236 (57.6)
Unknown	27 (6.6)
Cytoplasmic AR	409
IRS ≤ 2	282 (68.8)
IRS > 2	127 (31.0)
Missing	1 (0.2)
Nuclear AR	410
IRS ≤ 2	96 (23.4)
IRS > 2	314 (76.6)
Cytoplasmic AR-V7	406
IRS ≤ 2	59 (14.4)
IRS > 2	347 (84.6)
Missing	4 (1.0)
Granular AR-V7	405
< 5%	261 (63.7)
≥ 5%	144 (35.1)
Missing	5 (1.2)
OS	410
Alive	342 (83.4)
Dead	68 (16.6)
DSS	410
Yes	387 (94.4)
No	23 (5.6)
RFS	410
Yes	366 (89.3)
No	44 (10.7)

**Table 2:** Kaplan-Meier Analysis: Association of AR-V7 cytoplasmic staining and AR-V7 cytoplasmic granular staining with RFS.

Patient/Parameter	AR-V7 Cytoplasmic	RFS		AR-V7 Cytoplasmic Granular	RFS	
	IRS > 2 vs. ≤2			<5% vs. ≥5%		
	*N*	Months	*p*	N	Months	*p*
All patients	406	182.5 vs. 241.3	0.027	405	200.2 vs. 206.1	<0.001
Specific subsets						
Tumor stage pT2	233		n.s.	232	n.d.	<0.001
GS6	210		n.s.	209	187.4 vs. 200.2	0.040
Age ≤ 65 years	214		n.s.	213	194.2 vs. 199.1	0.001
Age > 65 years	192		(0.080)	192	181.3 vs. 204.9	0.045
Pn, no	145		n.s.	144	n.d.	0.005
Pn, yes	234		n.s.	234	170.2 vs. 194.5	0.018
CK20 IRS ≤ 2	299		(0.056)	299	170.2 vs. 179.2	0.001

Pn: perineural invasion; n.s: not significant; n.d: not determined (since all patients in the reference group did not experience relapse); p-values with a trend towards significance are in parenthesis.

**Table 3:** Univariate and multivariate Cox’s regression analyses: Association of AR-V7 cytoplasmic granular staining with RFS.

AR-V7 Granular ≥5% vs. <5%	Univariate Cox’s Regression Analysis			Multivariate Cox’s Regression Analysis (Adjusted for pT, GS and Pn)		
	N	RFS		N	RFS	
		HR (95% CI)	*p*		HR (95% CI)	*p*
All patients	405	7.2 (2.2–23.3)	0.001	355	5.3 (1.6–17.4)	0.006
Specific subsets						
Tumor stage pT2	232	53.1 (1.0–2731.1)	0.048	196		n.s.
GS6	209	6.6 (0.8-51.8)	(0.057)	195		n.s.
Age ≤ 65 years	213	13.5 (1.8–100.1)	0.011	189	9.7 (1.2–74.2)	0.029
Age > 65 years	192	3.9 (0.9-17.3)	(0.064)	166	3.8 (0.9–16.6)	(0.079)
Pn, no	144		n.s. ^1^	138		n.s. ^1^
Pn, yes	234	3.7	0.028	216	3.7 (1.1–12.1)	0.034
CK20 IRS ≤ 2	299	8.1 (1.2–3.1)	0.004	264	7.0 (1.7–29.8)	0.008

Pn: perineural invasion; n.s: not significant; p-values with a trend towards significance are in parentheses. ^1^ cannot be calculated since all patients with AR-V7 granular (≥5%) had no recurrence.

## Supplementary Materials

The following are available online at https://www.mdpi.com/2072-6694/12/9/2639/s1. Figure S1: Kaplan-Meier analysis: Combination of AR cytoplasmic staining and AR-V7 cytoplasmic staining and its association with RFS, Table S1: Detailed staining results for AR and AR-V7, Table S2: Bivariate correlations between immunohistochemical staining for AR/AR-V7 and clinicopathological and molecular parameters, Table S3: Univariate Cox’s regression analysis: Association of clinicopathological and molecular parameters with RFS.

## Abbreviations

AR	Androgen receptor
AR-V7	Androgen receptor splice variant 7
CK20	Cytokeratin 20
DSS	Disease-specific survival
GP88	Progranulin
GS	Gleason score
HR	Hazard ratio
IHC	Immunohistochemistry
IRS	Immunoreactive score
OS	Overall survival
PCa	Prostate carcinoma
Pn	Perineural invasion
RFS	Relapse-free survival
TMA	Tissue micro array
