# Supplementary material for: Expression of AR-V7 (Androgen Receptor Variant 7) Protein in Granular Cytoplasmic Structures Is an Independent Prognostic Factor in Prostate Cancer Patients
Source: Cancers (Basel). 2020 Sep 16;12(9):2639. doi: 10.3390/cancers12092639 (PMC7564112; doi:10.3390/cancers12092639)
Supplement: Supplementary file 1 [file cancers-12-02639-s001.pdf]

Supplementary Materials: Expression of AR-V7 (Androgen Receptor Variant 7) Protein in Granular Cytoplasmic Structures Is an Independent Prognostic Factor in Prostate Cancer Patients

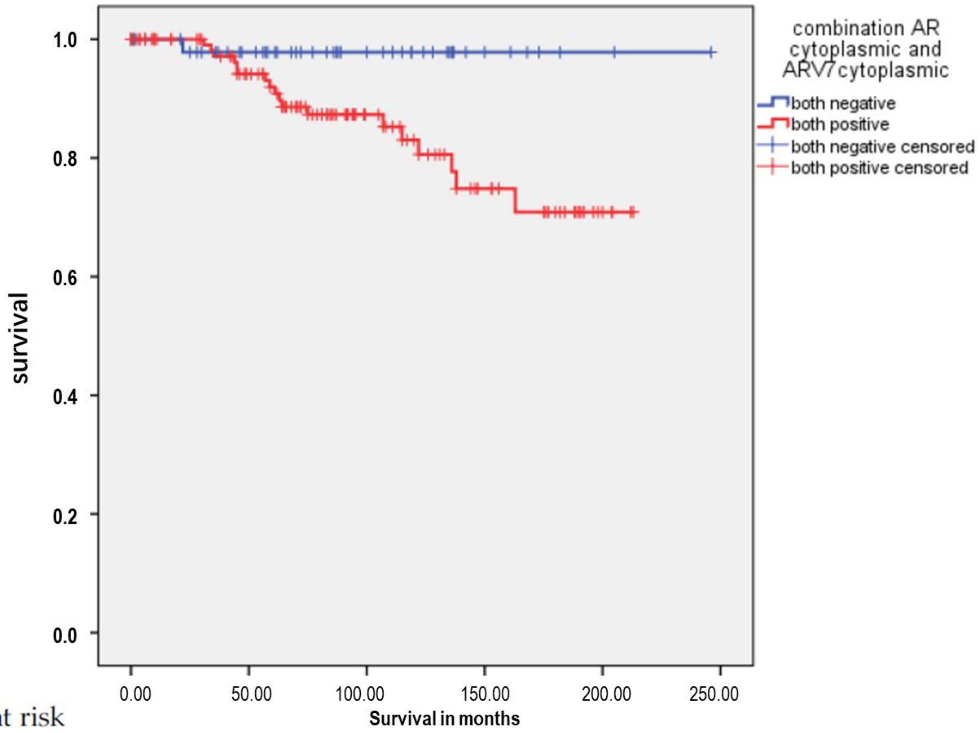

Number at risk						
both neg.	55	37	21	6	2	0
both pos.	120	90	44	22	4	0

Figure S1. Kaplan-Meier analysis: Combination of AR cytoplasmic staining and AR-V7 cytoplasmic staining and its association with RFS. Combination of AR cytoplasmic and AR-V7 cytoplasmic protein expression at comparing both positive vs both negative was associated with RFS (p = 0.025, log rank test).

Table S1. Detailed staining results for AR and AR-V7.

	**AR Cytoplasm**				**AR Nuclear**				**AR-V7 Cytoplasm**				**AR-V7 Granular**		
	IRS	Frequency	Percentage		IRS	Frequency	Percentage		IRS	Frequency	Percentage		Percentage per TCs	Frequency	Percentage of all
	0.00	120.00	29.3		0.00	38.00	9.30		0.00	14.00	3.40		0.00	260.00	63.40
	0.67	2.00	0.5		0.67	1.00	0.20		0.67	1.00	0.20		2.50	1.00	0.20
	1.00	50.00	12.2		1.00	12.00	2.90		1.00	6.00	1.50		5.00	1.00	0.20
	1.33	2.00	0.5		1.33	1.00	0.20		1.50	5.00	1.20		10.00	41.00	10.00
	1.50	11.00	2.7		1.50	10.00	2.40		2.00	33.00	8.00		15.00	2.00	0.50
	2.00	97.00	2..7		2.00	34.00	8.30		2.50	8.00	2.00		20.00	37.00	9.00
	2.33	1.00	0.2		2.33	1.00	0.20		3.00	39.00	9.50		25.00	1.00	0.20
	2.50	23.00	5.60		2.50	12.00	2.90		3.50	12.00	2.90		30.00	25.00	6.10
	3.00	54.00	13.20		2.67	1.00	0.20		4.00	69.00	16.80		40.00	11.00	2.70
	3.50	11.00	2.70		3.00	43.00	10.50		4.50	8.00	2.00		45.00	1.00	0.20
	3.67	1.00	0.20		3.50	11.00	2.70		5.00	28.00	6.80		50.00	8.00	2.00
	4.00	23.00	5.60		4.00	74.00	18.00		5.33	2.00	0.50		60.00	9.00	2.20
	4.50	2.00	0.50		4.50	11.00	2.70		5.50	3.00	0.70		70.00	4.00	1.00
	5.00	2.00	0.50		5.00	28.00	6.80		6.00	66.00	16.10		80.00	3.00	0.70
	5.50	2.00	0.50		5.33	1.00	0.20		6.50	1.00	0.20		90.00	1.00	0.20
	6.00	8.00	2.00		5.50	2.00	0.50		7.00	16.00	3.90	sum		405.00	98.80
sum		409.00	99.80		6.00	72.00	17.60		7.50	8.00	2.00	missing		5.00	1.20
missing		1.00	0.20		6.50	3.00	0.70		7.67	2.00	0.50	all		410.00	100.00
all		410.00	100.00		7.00	8.00	2.00		8.00	31.00	7.60				
					7.33	1.00	0.20		8.50	7.00	1.70				
					7.50	8.00	2.00		8.67	1.00	0.20				
					8.00	18.00	4.40		9.00	24.00	5.90				
					8.50	2.00	0.50		10.00	3.00	0.70				
					9.00	16.00	3.90		10.50	7.00	1.70				
					10.00	1.00	0.20		12.00	12.00	2.90				
					12.00	1.00	0.20	sum		406.00	99.00				
				sum/all		410.00	100.00	missing		4.00	1.00				
								all		410.00	100.00				

Table S2. Bivariate correlations between immunohistochemical staining for AR/AR-V7 and clinic-pathological and molecular parameters.

			**AR IRS Nuclear**	**AR IRS Cytoplasmic**	**AR-V7 IRS Cytoplasmic**	**AR-V7 Granular Percent**
**Spearman-Rho**	AR IRS nuclear	Correlation coeffient (Cc)	1.000	0.652**	0.401**	−0.109*
		Sig. (2-sided)		**<0.001**	**<0.001**	0.028
		N	410	409	406	405
	AR IRS cytoplasmic	Cc	0.652**	1.000	0.482**	−0.081
		Sig. (2-sided)	**<0.001**		**<0.001**	0.102
		N	409	409	405	404
	AR-V7 IRS cytoplasmic	Cc	0.401**	0.482**	1.000	−0.173**
		Sig. (2-sided)	**<0.001**	**<0.001**		**<0.001**
		N	406	405	406	405
	AR-V7 granular percent	Cc	−0.109*	−0.081	−0.173**	1.000
		Sig. (2-sided)	0.028	0.102	**<0.001**	
		N	405	404	405	405
	Age at diagnosis	Cc	−0.002	0.011	0.056	−0.014
		Sig. (2-sided)	0.971	0.832	0.261	0.776
		N	410	409	406	405
	pT	Cc	0.102*	0.160**	.152**	−0.169**
		Sig. (2-sided)	0.038	**0.001**	**0.002**	**0.001**
		N	410	409	406	405
	pN	Cc	−0.026	0.077	.116*	−0.125*
		Sig. (2-sided)	0.600	0.125	0.021	0.012
		N	402	401	398	397
	pM	Cc	0.043	0.073	0.051	0.025
		Sig. (2-sided)	0.400	0.152	0.320	0.631
		N	390	389	386	385
	Lymph invasion	Cc	0.000	0.070	0.143**	−0.044
		Sig. (2-sided)	0.993	0.200	0.009	0.421
		N	335	334	332	332
	Vaskular invasion	Correlation coeffient	0.067	−0.041	−0.047	−0.024
		Sig. (2-sided)	0.178	0.411	0.353	0.626
		N	403	402	399	398
	GS at prostatectomy	Cc	0.076	0.146**	0.167**	−0.147**
		Sig. (2-sided)	0.140	0.004	**0.001**	0.004
		N	381	380	377	376
	Perineural invasion	Cc	0.112*	0.142**	0.271**	−0.187**
		Sig. (2-sided)	0.028	0.005	**<0.001**	**<0.001**
		N	383	382	379	378
	Follow-up time RFS	Cc	0.081	0.094	0.062	−0.013
		Sig. (2-sided)	0.100	0.058	0.214	0.789
		N	410	409	406	405
	RFS	Cc	0.087	0.122*	0.083	−0.204**
		Sig. (2-sided)	0.079	0.014	0.094	**<0.001**
		N	410	409	406	405
	Follow-up time OS/DSS	Cc	0.094	0.122*	0.083	−0.073
		Sig. (2-sided)	0.056	0.013	0.096	0.142
		N	410	409	406	405
	OS	Cc	0.036	0.048	−0.040	−0.052
		Sig. (2-sided)	0.464	0.334	0.423	0.298
		N	410	409	406	405
	DSS	Cc	0.067	0.057	0.027	−0.059
		Sig. (2-sided)	0.178	0.253	0.594	0.232

	N	410	409	406	405
Follow-up time metastasis	Cc	0.075	0.118*	0.050	−0.046
	Sig. (2-sided)	0.131	0.017	0.319	0.357
	N	410	409	406	405
Metastasis	Cc	0.059	0.088	0.092	−0.173**
	Sig. (2-sided)	0.231	0.076	0.063	**<0.001**
	N	410	409	406	405
CK20 IRS cytoplasmic	Cc	0.222**	0.154**	0.116*	0.029
	Sig. (2-sided)	**<0.001**	**0.002**	0.019	0.556
	N	409	408	405	404

*. Correlation is on the 0.05 level significant (2-sided). **Correlation is on the 0.01 level significant (2-sided). Bonferroni correction α = 0.0025 (since 20 parameters: 0.05/20 = 0.0025). Abbreviations: Cc-correlation coefficient; Sig.-significance, AR-androgen receptor; pT-pathological tumor stage; pathological nodal stage; pM-pathological metastasis stage; RFS-relapse free survival, OS-overall survival; DSS-disease-specific survival; IRS-immunoreactive score.
Values below 0.0025 are in bold.

Table S3. Univariate Cox's regression analysis: Association of clinicopathological and molecular parameters with RFS.

Parameter	univariate Cox's regression analysis		
	**N**	**RFS**	
		HR	*p*
	410		
>65 years vs. ≤65 years	194 vs. 216	1.18	0.581
Pathological tumor stage	410		
pT2 vs. pT3+4	174 vs. 236	1.37	**0.041**
Gleason sum at prostatectomy	410		
GS6	213	reference	
GS7	108	5.02	**<0.001**
GS8-10	60	4.85	**<0.001**
Perineural invasion	383		
Pn positive vs. Pn negative	236 vs. 147	2.44	**0.013**
CK20	408		
negative vs. positive	303 vs. 105	1.25	0.556
AR cytoplasm	409		
IRS > 2 vs. IRS ≤2	127 vs. 282	1.32	0.370
AR nuclear	410		
IRS > 2 vs. IRS ≤2	314 vs. 96	1.39	0.417
AR-V7 cytoplasm	406		
IRS > 2 vs. IRS ≤2	347 vs. 59	6.87	0.057

Values $p < 0.05$ are in bold.

© 2020 by the authors. Licensee MDPI, Basel, Switzerland. This article is an open access article distributed under the terms and conditions of the Creative Commons Attribution (CC BY) license (http://creativecommons.org/licenses/by/4.0/).
